# Targeting Myeloid-Derived Suppressor Cells in Cancer Immunotherapy

**DOI:** 10.3390/cancers12092626

**Published:** 2020-09-15

**Authors:** Yufei Wang, Anna Jia, Yujing Bi, Yuexin Wang, Qiuli Yang, Yejin Cao, Yan Li, Guangwei Liu

**Affiliations:** 1 Key Laboratory of Cell Proliferation and Regulation Biology, Ministry of Education, Institute of Cell Biology, College of Life Sciences, Beijing Normal University, Beijing 100875, China; 201821200044@mail.bnu.edu.cn (Y.W.); 201831200019@mail.bnu.edu.cn (A.J.); 201931200019@mail.bnu.edu.cn (Y.W.); 201921200030@mail.bnu.edu.cn (Q.Y.); 201511200152@mail.bnu.edu.cn (Y.C.); liyan1106369@163.com (Y.L.); 2 State Key Laboratory of Pathogen and Biosecurity, Beijing Institute of Microbiology and Epidemiology, Beijing 100071, China; byj7801@sina.com

**Keywords:** myeloid-derived suppressor cells, regulatory T cells, immunosuppression, tumor microenvironment, therapy, cancer, tumor, immunotherapy, chemotherapy, radiotherapy

## Abstract

**Simple Summary:**

Myeloid-Derived Suppressor Cells (MDSCs) have been regarded as the main promoters of cancer development in recent years. They can protect tumor cells from being eliminated by neutralizing the anti-tumor response mediated by T cells, macrophages and dendritic cells (DCs). Therefore, different treatment methods targeting MDSCs, including chemotherapy, radiotherapy and immunotherapy, have been developed and proven to effectively inhibit tumor expansion. Herein, we summarize the immunosuppressive role of MDSCs in the tumor microenvironment and some effective treatments targeting MDSCs, and discuss the differences between different therapies.

**Abstract:**

Myeloid-derived suppressor cells (MDSCs), which are activated under pathological conditions, are a group of heterogeneous immature myeloid cells. MDSCs have potent capacities to support tumor growth via inhibition of the antitumoral immune response and/or the induction of immunosuppressive cells. In addition, multiple studies have demonstrated that MDSCs provide potential therapeutic targets for the elimination of immunosuppressive functions and the inhibition of tumor growth. The combination of targeting MDSCs and other therapeutic approaches has also demonstrated powerful antitumor effects. In this review, we summarize the characteristics of MDSCs in the tumor microenvironment (TME) and current strategies of cancer treatment by targeting MDSCs.

## 1. Introduction

The tumor microenvironment (TME) is a complex immune network that is a vital contributor to the promotion of tumor cell proliferation, metastasis, and immune escape. In the TME, other cells are present in addition to tumor cells, such as fibroblasts, immune and inflammatory cells, adipose cells, and immunosuppressive cells. In the TME, tumor cells incapacitate immune cells, including natural killer (NK) cells and T cells, by themselves and by immunosuppressive cells that are reprogrammed such that the tumor cells are not recognized and killed by the immune system. These “assistants” that assist tumorigenesis consist of tumor-associated macrophages (TAMs), regulatory T cells (T_regs_), cancer-associated fibroblasts (CAFs), and myeloid-derived suppressor cells (MDSCs). All members of these suppressive cells secrete large amounts of cytokines, chemokines, and other small molecule metabolites to build a hotbed suitable for the survival of malignant tumors [1,2,3].

**Figure 1:**
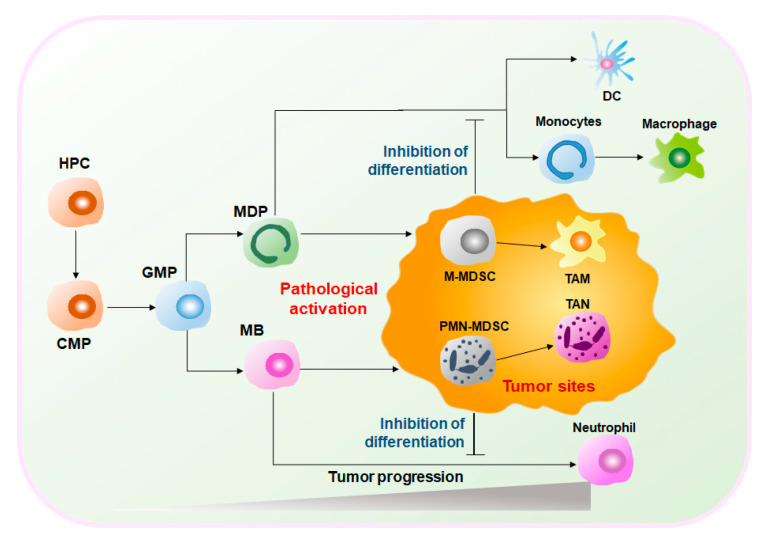
Differentiation and development of myeloid-derived suppressor cells (MDSCs) in the tumor microenvironment (TME). Under physiological conditions, neutrophils, dendritic cells (DCs), and monocytes originate from hematopoietic progenitor cells (HPCs) in the bone marrow. HPCs differentiate into granulocyte-macrophage progenitors (GMPs) after common myeloid progenitors (CMPs), and then GMPs differentiate into monocyte/macrophage and dendritic cell precursors (MDPs) and myeloblasts (MBs). Among them, MDPs are the precursors of DCs and monocytes, and MBs are the precursors of neutrophils. However, under pathological conditions, such as cancer, myeloid cells are induced to differentiate into suppressor cells, including monocytic myeloid-derived suppressor cells (M-MDSCs), tumor-associated macrophages (TAMs), polymorphonuclear myeloid-derived suppressor cells (PMN-MDSCs), and tumor-associated neutrophils (TANs). TME, tumor microenvironment; HPCs, hemopoietic progenitor cells; CMPs, common myeloid progenitors; GMPs, granulocyte-macrophage progenitors; MBs, myeloblasts; MDPs, monocyte/macrophage and dendritic cell precursors; M-MDSCs, monocytic myeloid-derived suppressor cells; PMN-MDSCs, polymorphonuclear myeloid-derived suppressor cells; TAMs, tumor-associated macrophages; TANs, tumor-associated neutrophils; DCs, dendritic cells.

MDSCs are a heterogeneous group of cells. Under normal circumstances, MDSCs represent a group of immature myeloid cells (IMCs) derived from bone marrow (BM) of various stages of differentiation and eventually differentiate into macrophages, dendritic cells (DCs), and neutrophils [4]. Therefore, MDSCs have considerable plasticity and diversity. However, under pathological conditions, such as the graft-versus-host disease (GVHD), autoimmune diseases, infections, and cancers, MDSCs are abnormally generated and activated [5]. Especially in the TME, hematopoietic progenitor cells (HPCs) are stimulated by tumor-derived inflammatory factors, e.g., granulocyte-macrophage colony-stimulating factors (GM-CSF), tumor necrosis factor-alpha (TNFα), vascular endothelial growth factor (VEGF), and prostaglandin E2 (PGE2), and differentiate into common myeloid progenitors (CMPs) and granulocyte-macrophage progenitors (GMPs). GMPs differentiate into monocyte/macrophage and dendritic cell precursors (MDPs) and myeloblasts (MBs) and are ultimately converted into MDSCs [6,7] (Figure 1). Activated MDSCs flow through the blood and spleen and are eventually recruited to the tumor site by C–X–C motif chemokine ligand 1 (CXCL1), C–C motif chemokine ligand 2 (CCL2), and other chemokines. MDSCs expressing anti-inflammatory factors such as interleukin (IL)-10 and transforming growth factor-beta (TGFβ) play important immunosuppressive roles in the TME to promote tumor development and expansion [6,8,9]. Given the obvious protumoral capabilities, tumor treatment strategies targeting MDSCs are highly valued. In this review, we summarize the classification of MDSCs, their functional characteristics in the TME and how MDSCs exert immunosuppressive functions. On the other hand, we discuss cancer treatments by targeting MDSCs and combination therapy of immunotherapy and targeting MDSCs.

## 2. MDSCs in the TME

### 2.1. Classification and Functional Differences of MDSCs

The identification of MDSCs has been controversial. Since the phenotype and morphology of MDSCs are similar to those of neutrophils and monocytes, the distinction between MDSCs and these cells is unclear. In mice, MDSCs mainly include polymorphonuclear MDSCs (PMN-MDSCs) and monocytic MDSCs (M-MDSCs). Among them, M-MDSCs are defined as CD11b^+^Ly6C^+^Ly6G^−^; conversely, PMN-MDSCs are defined as CD11b^+^Ly6C^−/low^Ly6G^+^. In humans, MDSCs consist of M-MDSCs, PMN-MDSCs, and early-MDSCs (e-MDSCs). M-MDSCs have the phenotype CD11b^+^CD33^+^CD14^+^CD15^−^HLA-DR^−/low^, whereas PMN-MDSCs are phenotypically CD11b^+^CD33^+^HLA-DR^−/low^CD14^−^CD15^+^ (or CD66b^+^) [10,11,12]. Given the similar phenotype to neutrophils, studies have shown that lectin-type oxidized LDL receptor 1 (LOX-1) is expressed in PMN-MDSCs in the peripheral blood and tumor sites of cancer patients, so it can be distinguished based on the expression of LOX-1 [13]. e-MDSCs, which are composed of immature MDSC progenitor cells, are defined as HLA-DR^-^CD33^+^Lin^−^(CD3^−^CD14^−^CD15^−^CD19^−^CD56^−^) [6,14]. Nevertheless, it is still necessary to continue to explore methods to distinguish MDSCs from other immune cells although the strength of the immunosuppressive effect on T cells is typically used to identify MDSCs.

There are differences in the mechanisms by which two MDSC subtypes exert inhibitory functions. M-MDSCs highly express inducible nitric oxide synthase (iNOS, also known as NOS2) through the signal transducer and activator of transcription (STAT1) signaling pathway, generating large amounts of nitric oxide (NO), whereas PMN-MDSCs produce high levels of reactive oxygen species (ROS) and less NO through the STAT3 pathway to suppress immune responses [15]. Both subtypes overexpress arginase 1 (ARG1), and both iNOS and ARG1 can reduce the concentration of arginine in the microenvironment and promote T cell apoptosis [4,16,17,18]. In general, both M-MDSCs and PMN-MDSCs have the potential to inhibit the antitumor response, but the mechanism is not identical.

### 2.2. Immunosuppressive Activity of MDSCs in the TME

**Figure 2:**
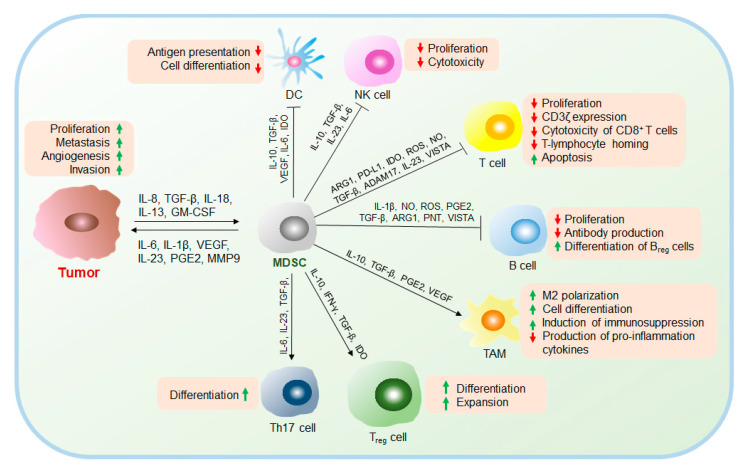
Multiple mechanisms of immunosuppression mediated by MDSCs in the TME. MDSCs secrete a large number of cytokines to remodel the TME by accelerating tumor development, angiogenesis, and metastasis; inhibiting the antitumor response mediated by T cells, B cells, NK cells, and DCs; and promoting the differentiation of immunosuppressive TAMs, T_regs_, and Th17 cells. NK cells, natural killer cells; T_regs_, regulatory T cells; B_regs_, regulatory B cells.

The immunosuppressive function of MDSCs is a double-edged sword, and very different roles are noted under different circumstances [18] (Figure 2). For example, in organ transplantation, high levels of MDSCs inhibit CD8^+^ T cell-mediated graft-versus-host disease (GVHD) and translate to better graft survival [19,20,21]. However, in cancer, MDSCs are major contributors to tumorigenesis, metastasis, and development [22] (Figure 2). MDSCs significantly inhibit the antitumor activity of T cells, especially cytotoxic T lymphocytes (CTLs), and also make proinflammatory cells, such as NK cells, DCs, and B cells incompetent; in addition, MDSCs induce the generation of anti-inflammatory T_regs_, TAMs, and Th17 cells, which remodel the microenvironment that supports tumor development [23]. MDSCs promote tumor epithelial–mesenchymal transformation (EMT) by expressing factors, such as TGFβ, IL-6, hepatocyte growth factor (HGF), and high mobility group binding (HMGB)-1, which makes tumors exhibit aggressive phenotypes with high migration ability [16]. In addition, MDSCs establish a premetastatic niche (pMN) for tumor development [24]. MDSCs also facilitate angiogenesis by secreting matrix metalloprotease 9 (MMP9), which induces VEGF release from the matrix [25]. Moreover, MDSCs subsequently promote tumor mesenchymal–epithelial transition (MET) to facilitate cancer cell proliferation [26].

#### 2.2.1. Suppression of T cells

MDSCs inhibit the activity of T cells through multiple mechanisms. First, amino acids are essential nutrients for T cell proliferation and activation. MDSCs catalyze the decomposition of L-arginine into urea and L-ornithine (Orn) or NO and L-citrulline by overexpressing ARG1 and iNOS and increase the uptake of L-arginine by elevating the expression of cationic amino acid transporter 2 (CAT2) [27,28]. L-arginine is an important part of the T cell receptor (TCR) ζ-chain. The depletion of L-arginine caused by MDSCs prevents T cells from recognizing antigens and further causes T cells to remain in the G_0_/G_1_ cell cycle, suppressing the immune response [29]. MDSCs make use of the transporter solute carrier family 7 member 11 (SLC7A11) to sequester cysteine. T cells cannot synthesize cysteine autonomously and can only take up the cysteine delivered by DCs [30]. Therefore, MDSCs inhibit T cell activation and induce apoptosis by reducing the cysteine concentration in the TME [31]. L-tryptophan is also an important nutrient required for T cell proliferation. MDSCs overexpress indoleamine 2,3-dioxygenase (IDO) in an IL-6-dependent manner to convert L-tryptophan to kynurenine (Kyn) by stimulating the STAT3 signaling pathway. Thus, T cell proliferation is inhibited and vascularization is promoted [32,33]. Second, MDSCs produce a large amount of ROS and nitrogen species (RNS) by means of NADPH oxidase (NOX), ARG1, and iNOS, which inhibit the synthesis of the TCRζ-chain and further cause T cell anergy [8,34]. Third, MDSCs interact with the L-selectin (CD62L) molecule on the surface of T cells through ADAM metallopeptidase domain 17 (ADAM17), which reduces the homing of T cells to peripheral lymph nodes (PLNs) [35]. Fourth, immune checkpoints are widely believed to negatively regulate T cell activation. Programmed death ligand-1 (PD-L1) on MDSCs induced by hypoxia-inducible factor 1-alpha (HIF-1α) promotes apoptosis of T cells by binding to programmed cell death-1 (PD-1) on the surface of T cells [36]. Fifth, V-domain Ig suppressor of T cell activation (VISTA) was recently identified and is expressed on MDSCs and negatively regulates CD8^+^ T cells [37,38]. Sixth, MDSCs attenuate the development of tumor antigen-specific effector T-helper cells (Th1) through IL-6 production [39]. Recently, a report demonstrated that methylglyoxal, a metabolite of MDSCs, induces T cell paralysis, which can be overcome by neutralizing the dicarbonyl activity in methylglyoxal [40]. In human early-stage lung cancer, a subset of tumor-associated neutrophils (TANs) similar to antigen-presenting cells (APCs) does not exhibit immunosuppressive effects but instead promotes the proliferation and activation of T cells and enhances the antitumor response [41,42,43,44,45]. Whether PMN-MDSCs with similar phenotypes can trigger the antitumor effect of T cells in the early stage of tumorigenesis is also worthy of attention.

#### 2.2.2. Inhibition of NK cells, DCs, and B cells

It has been reported that MDSCs can inhibit NK cells, DCs, and B cells. MDSCs inhibit NK cell cytotoxicity, downregulate the NK activation-related receptor NKG2D by secreting TGFβ, and reduce interferon gamma (IFNγ) production. IL-23 produced by myeloid cells can effectively inhibit NK cells. After blocking IL-23R, the proportion of NK cells expressing IFNγ increased significantly [46]. Secreted IL-6 inhibits the proliferation and activation of NK cells. Moreover, the production of IL-10 by MDSCs also affects the function of NK cells [1]. Treatment with the MDSC inhibitor SX-682 significantly improved the antitumor effect of NK cells [47]. MDSCs antagonize NK-cell Fc receptor-mediated functions, including cytokine production, signal transduction, and antibody-dependent cellular cytotoxicity, in a contact-independent manner via iNOS-dependent NO production [48].

In the TME, activated HIF-1α stimulates the expression of VEGF in MDSCs, which inhibits the differentiation of DCs [48]. In addition, IL-10 produced by MDSCs impairs DC cell activation and proinflammatory functions by suppressing the production of IL-12 in DCs and inhibiting the T cell stimulatory activity of DCs [28,49]. MDSCs reduce the maturation of DCs in a dose-dependent manner and damage the ability of DCs to take up antigens, migrate, and induce T cells to produce IFNγ [9]. MDSCs prevent the differentiation of DCs, thereby further increasing the accumulation of MDSCs through STAT3-mediated activation of the expression of calcium binding proteins S100A8/A9 [15]. The activated STAT3 signal in MDSCs induces the expression of NOX2, thereby generating a large amount of ROS to prevent the differentiation of DCs [1,49].

Another cell type that interacts with MDSCs is B cells. IL-7 and downstream STAT5 signaling pathways play a key role in the development and differentiation of B cells, but these pathways are damaged during tumor development; in addition, the decrease in serum immune globulin (Ig)G levels indicates impaired B cell function [50]. After the elimination of MDSCs with anti-Gr-1 antibody, serum IgG and IL-7 levels recovered, and the level of TGFβ1 decreased, indicating that MDSCs negatively regulate the immune response of B cells. Moreover, MDSCs also inhibit B cell proliferation in vitro in an ARG1-dependent manner [50]. The accumulation of IgA^+^ B cells expressing PD-L1 and IL-10 can be promoted by MDSCs in a TNF receptor 2 (TNFR2)-dependent manner [51,52]. Due to the exclusive loss of L-selectin through a contact-dependent mechanism and interaction with ADAM17 expressed by MDSCs, the homing of B cells to distant lymph nodes is disrupted [53]. MDSCs also serve as inducers of the differentiation of regulatory B cells (B_regs_) by secreting inflammatory cytokines, such as IL-10 and TGFβ [54]. The proliferation of B cells activated by IL-4 and LPS is inhibited by MDSCs in a T cell-dependent or T cell-independent manner [54]. Similar to T_regs_, B_regs_ also mediates suppression of the antitumor response by inducing T cell apoptosis [55]. In the 4T1 breast cancer model, MDSCs seem to induce the accumulation of B_regs_ with high PD-L1, thereby producing IL-10 and IgA and inhibiting T cells by inducing apoptosis [54].

#### 2.2.3. Induction of T_regs_

The accumulation of immunosuppressive T_regs_ is also a major contributor to tumor invasion and expansion, similar to MDSCs. MDSCs promote the skewing of CD4^+^ T cells into T_regs_ [56]. The secretion of TGFβ, IL-10, and IFNγ has been implicated in the differentiation and activation of T_regs_ [57,58]. MDSCs exert immunosuppressive functions by mediating the induction and recruitment of T_regs_, in which MDSCs expressing CD40 interact with CD40L on T_regs_ [59]. Another mechanism by which MDSCs mobilize T_regs_ involves promoting the migration of T_regs_ to tumor sites and lymph nodes through overexpression of IDO [60,61].

#### 2.2.4. Activation of TAMs and Th17 cells

MDSCs are also involved in the activation of TAMs and Th17 cells. MDSCs promote the transformation of M1 macrophages into M2 TAMs [10]. TAMs are reprogrammed to reduce IL-12 production and increase IL-10 release, resulting from the interaction with MDSCs [62]. Hypoxia in the TME induces the downregulation of STAT3 activity in MDSCs, thereby promoting the differentiation of M-MDSCs into TAMs [63]. IL-17-producing T helper cells (Th17) suppress the antitumor activity of T cells [64]. The production of cytokines IL-1β, IL-6, IL-23, and TGFβ in MDSCs activates the expression of iNOS in T cells, which promotes the differentiation and proliferation of Th17 cells [54,65].

#### 2.2.5. Exosome

Exosomes represent an abundant group in the TME. MDSCs secrete exosomes that are packed with a large number of tumor-promoting factors to exert immunosuppressive effects. Proteins, such as TGFβ, MMP, IL-10, and S100A8/A9, and microRNA are transported by exosomes and play an important role in inducing MDSCs to exert immunosuppressive functions, promoting angiogenesis, and promoting tumor metastasis [55,63].

Overall, the TME is a complex network composed of diverse immune cells. MDSC-mediated immune suppression occurs through multiple mechanisms. If we want to dynamically and deeply study the role of MDSCs in the TME, comprehensive research is necessary.

## 3. The Therapeutic Effects of Targeting MDSCs

**Figure 3:**
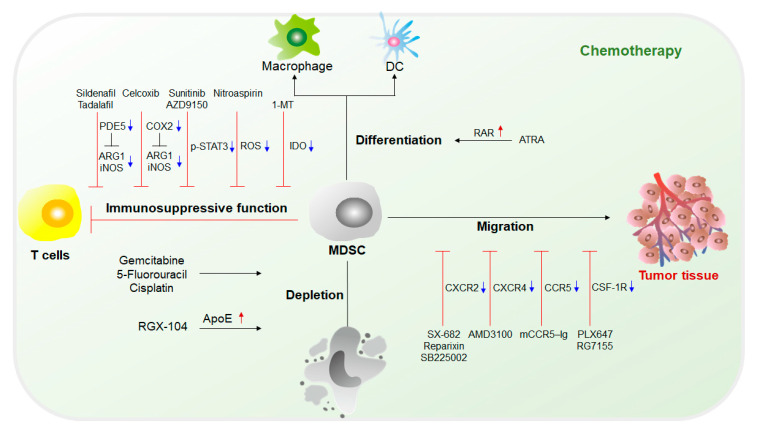
Chemotherapy targeting MDSCs. Current chemotherapeutics targeting MDSCs are mainly studied from four aspects: (1) Attenuation of the immunosuppressive activity of MDSCs by downregulating the expression of ARG1, iNOS, and IDO, the activation of STAT3 and the production of reactive oxygen species (ROS); (2) induction of MDSC differentiation inducing MDSCs to differentiate into mature myeloid cells, such as DCs and macrophages, to initiate and regulate immune responses; (3) targeting chemokine receptors on the surface of MDSCs to prevent MDSCs from migrating to tumor tissues; and (4) promotion of MDSC deletion to reduce the population of MDSCs. ARG1, arginase 1; iNOS, inducible nitric oxide synthase; IDO, indoleamine 2,3-dioxygenase; STAT3, signal transducer and activator of transcription 3; ROS, reactive oxygen species; PDE5, phosphodiesterase 5; COX2, cyclooxygenase-2; ATRA, all-trans retinoic acid; RAR, retinoic acid receptor; CXCR, C-X-C chemokine receptor; CCR, CC chemokine receptor; CSF-1R, colony-stimulating factor-1 receptor.

**Figure 4:**
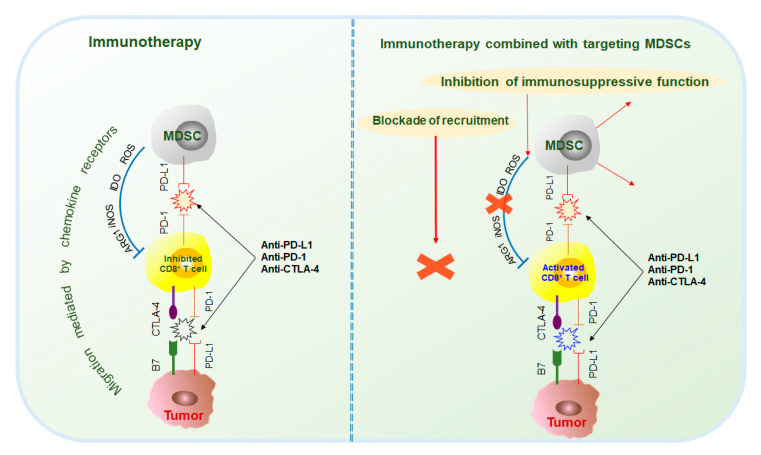
Combination of immunotherapy and targeting MDSCs. Using an immunotherapy regimen alone, the therapeutic outcome is not very satisfactory mainly due to the strong suppressive effect of MDSCs on cytotoxic T cells. Dual therapy involving immunotherapy and targeted MDSCs can enhance the therapeutic effect of immunotherapy. On one hand, it can effectively reduce the population of MDSCs; on the other hand, it can also greatly weaken the ability of MDSCs to inhibit cytotoxic T cells. PD-1, programmed death 1; PD-L1, programmed death ligand-1; CTLA-4; cytotoxic T lymphocyte-associated antigen 4; B7, costimulatory molecules.

Immunotherapy is currently the mainstream cancer therapy and can effectively save the lives of cancer patients through an immune checkpoint blockade (ICB) [66]. However, immunotherapy is not effective for every patient. Only a few patients can be cured, and it is limited to specific types of cancer. The immunosuppressive function of MDSCs is considered to make a major contribution to tumor development given their extensive inhibition of antitumor responses and promotion of tumorigenesis. Studies have shown that MDSCs are the main contributors to the poor clinical outcome of immunotherapy [67,68]. Therefore, in recent years, a variety of cancer treatment strategies have been developed to reduce the number of MDSCs and impede the immunosuppressive function of MDSCs. In addition, some traditional treatment approaches, such as radiotherapy or other methods, can also effectively damage the inhibitory activity of MDSCs [69,70]. Furthermore, a large number of studies have combined treatment methods targeting MDSCs with immunotherapy, which has exhibited potential antitumor effects (Figure 3 and Figure 4).

### 3.1. Chemotherapy Targeting MDSCs

The fundamental purpose of therapy targeting MDSCs is to eliminate MDSCs. Without the immunosuppression mediated by MDSCs, the limitation of the antitumor response can be lifted, and tumor development can be suppressed. Current chemotherapy approaches targeting MDSCs mainly include (1) inhibition of immunosuppressive functions of MDSCs; (2) elimination of MDSCs in both tumor sites and the circulatory system; (3) blockade of MDSC recruitment to the TME; and (4) induction of the differentiation of MDSCs into mature myeloid cells that lack suppressive activity [4,71,72] (Figure 3).

#### 3.1.1. Inhibition of Immunosuppressive Functions

Diminishing the protumoral effects of MDSCs can be achieved by weakening the immunosuppressive function of MDSCs.

As mentioned above, STAT3 plays an indispensable role in MDSC-mediated tumorigenesis. By applying a specific small molecule inhibitor of p-STAT3 or STAT3-targeted siRNA to block the activation of STAT3, the suppressive activity of MDSCs can be eliminated by reducing the expression of ARG1 in MDSCs [73,74]. Receptor tyrosine kinases, such as TYRO3 (a type of protein tyrosine kinase), AXL (a type of receptor tyrosine kinase), and C-Mer proto-oncogene tyrosine kinase (MERTK) and their ligands, Gas 6 and Protein S, can reverse the tumorigenic properties of MDSCs, increase the numbers of tumor infiltrating CD8^+^ T cells, and strengthen anti-PD-1 immune checkpoint therapy. MERTK abolishes the suppressive capability of MDSCs by negatively regulating STAT3 [75]. Moreover, all STAT3 inhibitors, such as sunitinib, AZD9150, and BBI608, or a conjugate of the STAT3 antisense oligonucleotide (ASO) tethered to immunostimulatory toll-like receptor 9 (TLR9) agonist (CpG-STAT3ASO) conjugates can significantly diminish the immunosuppressive function of MDSCs and rescue antitumor immunity [48,76,77,78].

PGE2 induces MDSCs to upregulate the production of ARG1 and iNOS and exert suppression. Cyclooxygenase-2 (COX-2) is the upstream molecular signal of PGE2, which regulates the generation of PGE2. Thus, COX-2 can be targeted to negatively regulate the synthesis of PGE2. shRNA targeting of COX-2 significantly reduces MDSCs in the spleens of tumor-bearing mice [79]. COX-2 expression can also be inhibited by acetylsalicylic acid, NS-398, and celecoxib, thereby hindering the activity of MDSCs and increasing the infiltration of CTLs in tumor sites [80,81,82]. RIPK3 induces cell necrosis by interacting with TLR3/4 [83]. RIPK3 deficiency activates the NF-κB signaling pathway and upregulates the expression of the downstream signaling molecules COX-2 and PGE2, which aggravates the immunosuppressive activity of MDSCs and accelerates tumor growth. Treatment with aspirin (ASA, COX inhibitor) significantly protected mice against tumorigenesis [84]. Additionally, the overexpression of fatty acid transport protein 2 (FATP2) is also involved in the synthesis of PGE2 through the activation of the STAT5 signaling pathway. Administration of the selective FATP2 inhibitor lipofermata selectively inhibits the function of MDSCs while enhancing immunotherapy [85].

Phosphodiesterase 5 (PDE5) is another target of MDSC treatment that is a hydrolase that acts on the NO/cyclic guanosine monophosphate (cGMP) signaling pathway [86]. The application of PDE5 inhibitors, including sildenafil, tadalafil, and vardenafil, can reduce the production of ARG1 and iNOS in MDSCs, abolish the inhibitory activity of MDSCs, reduce the number of T_regs_, and thus greatly delay the progression of tumors [87,88,89,90]. Treatment with tadalafil combined with cytokine-induced killer (CIK) cell-based immunotherapy enhanced CIK activity against human hepatocellular carcinoma (HCC) cell lines in vitro [91]. Nitroaspirin is another inhibitor of ARG1 and iNOS that reduces ROS generation [92].

Nuclear factor E2-related factor 2 (Nrf2), a transcription factor, is considered to be the main regulator of antioxidant stress. Nrf2 is associated with abnormal ROS accumulation in MDSCs, which has been confirmed by a model of Nrf2-deficient mice. In Nrf2 knockout (KO) mice, the circulating level of MDSCs did not change; however, with elevated amounts of cellular ROS, the number of CD8^+^ T cells was significantly reduced, and the tumor growth rate increased [93,94]. Treatment with Nrf2-inducing triterpenoids, such as omaveloxolone (RTA-408), CDDO-Me (RTA-402), and CDDO-Im (RTA-403), increases the transcriptional activity of Nrf2, which attenuates the production of ROS, abrogates the immune suppressive effect of MDSCs, and protects immune cells and tissues from oxidative stress [95,96,97]. However, a recent study has demonstrated that Nrf2 is activated by PKR-like endoplasmic reticulum (ER) kinase (PERK) in tumor-infiltrating MDSCs, giving MDSCs the potential for immunosuppression [98]. The deletion of PERK or treatment with the selective inhibitor of PERK AMG-44 reduces Nrf2 transcription, resulting in ROS overexpression, causing mitochondrial damage, impeding the immunosuppression of MDSCs, and increasing the infiltration of CD8^+^ T cells. This situation can be antagonized by the addition of the Nrf2 inducer sulforaphane [98]. According to the above report, it can be concluded that Nrf2overexpression and deletion affect the immunoinhibitory activity of MDSCs. Only when Nrf2 maintains a steady state can MDSCs exert normal protumor effects.

N-Hydroxy-nor-L-arginine (nor-NOHA) is used as an ARG1 inhibitor. Blocking ARG1 by nor-NOHA reversed the immunosuppressive activity of MDSCs [99]. Inhibition of the VEGF/VEGFR-2 axis with antibody DC101 repressed primary tumor growth and metastasis in the 4T1 breast cancer model. However, it had no effect on MDSC mobilization and induced ARG1 expression. Combination treatment with nor-NOHA and DC101 reduced the inhibitory effect of MDSCs, but T cell proliferation was inhibited [100]. 1-Methyl-DLtryptophan (1-MT), a competitive inhibitor of IDO, ablates the immunosuppressive function of MDSCs on T cells. When 1-MT is combined with nor-NOHA, the T cell proliferation rate is almost completely restored [101]. Bruton’s tyrosine kinase (BTK) is a nonreceptor intracellular kinase that is related to the migration and proliferation of MDSCs. Treatment with the BTK inhibitory drug ibrutinib decreases the cytokine production and motility of MDSCs [102].

Recently, it was described that estrogen interacts with estrogen receptor alpha, driving the mobilization of MDSCs by activating the STAT3 pathway, which facilitates deregulated myelopoiesis. The progression of tumors can be delayed by removing estrogen activity though an anti-estrogen treatment [103]. Castration-resistant prostate cancer exhibits resistance to androgen deprivation therapy mainly because IL-23 secreted by MDSCs activates the androgen receptor (AR) and the STAT3/RORγ signaling axis in prostate tumor cells. Blocking the production of IL-23 can counteract MDSC-mediated CRPC through treatment with the anti-IL-23 antibody and AR antagonist enzalutamide [104].

MDSCs have low glycolysis and mitochondrial respiratory capacity but contain high levels of methylglyoxal, which inhibits the antitumor activity of CD8^+^ effector T cells. Neutralization of methylglyoxal with compounds containing guanidine groups, such as metformin, can effectively abolish the immunosuppressive activity of MDSCs. The combination of metformin and anti-PD-1 overcomes the suppression of immunotherapy by MDSCs [40].

#### 3.1.2. Depletion of MDSCs

The most direct MDSC-targeting therapy strategy is to eliminate MDSCs. Treatment with low doses of chemotherapy drugs, such as gemcitabine, 5-fluorouracil (5-FU), paclitaxel, and cisplatin, effectively affects the viability of MDSCs [8,28,105]. Gemcitabine is a selective inhibitor of MDSCs that reduces the number of circulating T_regs_ and the level of TGFβ1 and PMN-MDSCs but not M-MDSCs in the peripheral blood of patients with pancreatic cancer and restores the proliferation and antitumor capacity of effector T cells [106]. 5-FU can equally induce the death of the two subtypes of MDSCs and has no obvious effect on other immune cells, such as T cells, NK cells, DCs, and B cells. Treatment with 5-FU triggered the apoptosis of MDSCs, promoted tumor-infiltrating T cells to produce high levels of IFNγ and enhanced the T cell-dependent antitumor response in the mouse EL4 model [107]. Therefore, compared with gemcitabine, 5-FU significantly and specifically eliminated MDSCs by inducing apoptosis in the TME and spleen of tumor-bearing mice [107]. However, the assembly of NLRP3 in MDSCs is activated by 5-FU, which leads to the secretion of MDSC-derived IL-1β and CD4^+^ T cell-derived IL-17 and inhibits the antitumor effect of 5-FU. As a countermeasure, the combination of 5-FU and IL-1β inhibitors, such as the indirect inhibitors DHA and SP600125, could represent a successful approach [108,109]. Docetaxel, which has the same effect as paclitaxel, was shown to significantly inhibit tumor growth. Docetaxel achieves its antitumor effect by polarizing MDSCs to M1-type macrophages, reducing the proportion of MDSCs in the spleen [110]. ApoE impedes tumor invasion and endothelial cell recruitment, but liver-X receptors (LXRs) inhibit ApoE expression. Recently, it has been reported that the LXR agonists GW3965 and RGX-104 impair MDSC survival by activating the LXR/ApoE axis and enhance the antitumor activity of CTLs [111,112]. CD33 is highly expressed on MDSCs in humans, especially M-MDSCs, but CD33 is a therapeutic target on circulating and tumor-infiltrating MDSCs across multiple cancer types [113]. The immunotoxin gemtuzumab ozogamicin, a CD33 monoclonal antibody (mAb), effectively eliminates MDSCs and reactivates T cells to fight against multiple cancers [113,114]. Additionally, targeting the bromodomain and extraterminal domain (BET), a component of the endogenous transcription enhancer of MDSCs, by treating HCC patient-derived PBMCs with the small molecular inhibitor i-BET762 significantly reduced the number of CD14^+^HLA-DR^−/low^ M-MDSCs and enhanced the effect of immunotherapy [115].

#### 3.1.3. Blockade of Migration

Blocking the migration of MDSCs can effectively reduce the proportion of MDSCs in the TME and the periphery by impeding the response of MDSCs to chemokines [2,22]. Antagonists of chemokines help prevent MDSCs, especially PMN-MDSCs, from reaching the tumor sites and modifying the immunosuppressive microenvironment [116]. CXCR2 is an important chemokine receptor for MDSC trafficking [117,118]. Blocking the CXCR2/CXCLs pathway through CXCR2 inhibitors, such as SX-682, reparixin, and SB225002, effectively reduces the infiltration of MDSCs and improves the function of cytotoxic T cells [84,119,120]. The progression and invasiveness of multiple tumors can be suppressed by targeting the CCR5/CCL axis [121,122,123,124]. Administration of mCCR5–Ig-neutralizing CCR5 ligands reduced the migration of MDSCs and T_regs_ without impacting the recruitment of effector T cells to the TME [125]. The CXCR4 receptor for CXCL12 (also known as stromal cell-derived factor 1, SDF-1) also mediates the recruitment of MDSCs. Neutralization of CXCR4 by antagonists, such as AMD3100, reduces the number of MDSCs and T_regs_ and promotes M2 to M1 macrophage polarization in the TME [126,127]. Moreover, the colony-stimulating factor-1 receptor (CSF-1R) is a tyrosine kinase receptor that, when combined with the receptor, can induce the formation of MDSCs and trafficking to tumor sites. It has recently been reported that CSF-1R inhibitors, such as RG7155 and PLX647, block the CSF-1R signaling pathway, leading to ablation of MDSCs or inhibition of their tumor-promoting functions and reprogramming of TAMs [68,128,129,130].

#### 3.1.4. Induction of Differentiation

Furthermore, there is another therapeutic method that targets MDSCs by inducing MDSCs to differentiate into cells with a proinflammatory phenotype. All-trans retinoic acid (ATRA) is a metabolic intermediate of vitamin A and has been identified as an anticancer drug that induces MDSCs to differentiate into DCs and macrophages [32,131,132]. ATRA induces the differentiation of MDSCs both in vivo and in vitro, which greatly reduces the number of MDSCs. The specific mechanism is that the added ATRA activates the ERK1/2 signal, which further upregulates the expression of glutathione synthase in MDSCs, resulting in increased glutathione levels, neutralization of the generated ROS, and inhibition of MDSC inhibitory activity [133]. Finally, myeloid cells differentiate in response to treatment with ATRA. It is worth noting that the effects of ATRA on MDSCs are highly time-dependent with tumor vaccination [134]. Evidence suggests that vitamin D3 may also promote the differentiation of MDSCs. MDSCs at the tumor site have higher levels of vitamin D receptor compared with those in the spleen and bone marrow. Treatment with the active form of vitamin D3 (1α,25-dihydroxyvitamin D3,1,25(OH)D) significantly reduced the T cell suppressive capacity of MDSCs. In vitro-derived MDSCs reduced the production of NO under the stimulation of 1,25(OH)D [135]. Another study reported that the addition of 1,25(OH)D abolished the accumulation of IL-6-induced MDSCs [136]. In summary, therapies targeting MDSCs reduce the number and function of MDSCs at tumor sites and the circulation. However, simply targeting MDSCs is difficult to achieve the goal of tumor elimination, and combined therapy can prevent tumor growth more efficiently.

### 3.2. Immunotherapy in Combination with MDSC Targeted Therapy

Tumor and immunosuppressive cells, such as MDSCs, also inhibit antitumor responses through the interaction of immune checkpoint molecules, such as PD-1/PD-L1, CTLA-4/B7, and Gal-9/TIM-3 [137]. Current studies mainly focus on the immunotherapy of PD-1, PD-L1, and CTLA-4. PD-1 antibodies include pembrolizumab and nivolumab; PD-L1 antibodies include atezolizumab, durvalumab, and avelumab; and CTLA-4 antibodies include ipilimumab and tremelimumab [137,138]. However, because MDSCs are the main contributors to immunosuppression, the effects of immunotherapy are often hindered. Therefore, the combination of immunotherapy and targeted MDSCs has been thoroughly researched and has made great progress (Figure 4).

#### 3.2.1. Immunotherapy Combined with Inhibition of Immunosuppressive Functions of MDSCs

FATP2 is overexpressed in PMN-MDSCs and promotes tumor development. Combined treatment with the FATP2 inhibitor lipofermata and checkpoint inhibitors anti-CTLA-4 or anti-CSF-1R inhibitors abolished the inhibitory activity of PMN-MDSCs and blocked tumor progression in mice [85]. BLZ945, a selective inhibitor of CSF-1R, enhances the response to anti-PD-1 treatment in neuroblastoma mice [139]. CB-1158, a small molecule inhibitor of ARG1, blocks MDSC-mediated suppression of T cells in vitro and in various mouse models of cancer. CB1158 or anti-PD-L1 monotherapy slows down the development of tumors, while treatments combining the two drugs enhance the inhibition of tumor growth [140].

#### 3.2.2. Immunotherapy Combined with Depletion of MDSCs

The combination therapy of SRA737, an oral CHK1 inhibitor, and anti-PD-L1 leads to the activation of antitumor effects. After adding low-dose gemcitabine to the combination therapy in the small cell lung cancer model, the number of antitumor CD8^+^ cytotoxic T cells, DCs, and M1 macrophages was more significantly increased. In addition, MDSCs and T_regs_ were decreased [141]. Additionally, when administered in combination with gemcitabine, immunotherapy can impair tumorigenesis and expansion mainly due to depletion of immunosuppressive cells, such as MDSCs [142]. In the mouse model of fibrotic liver, the accumulation of M-MDSCs rather than the accumulation of PMN-MDSCs is related to the increase in tumor infiltrating lymphocytes and tumorigenicity. Moreover, in human liver cancer, CD33^+^M-MDSCs are obviously enriched in the fibrotic liver near the tumor [115]. Combined therapy with anti-PD-L1 and BET bromodomain inhibitor i-BET762, which is currently in clinical trials, synergistically inhibited the suppressive function of M-MDSCs and enhanced tumor-infiltrating lymphocytes in a fibrotic-HCC mouse model [115].

#### 3.2.3. Immunotherapy Combined with a Blockade of Recruitment of MDSCs

Mutated KRAS gene (KRASG12D)-mediated suppression of IRF2 in colorectal cancer leads to increased secretion of CXCL3, thereby promoting the migration of MDSCs into tumor sites through interaction with CXCR2. Treatment of tumor-bearing mice with the CXCR2 inhibitor SX-682 and anti-CXCR2 antibodies decreased the migration of MDSCs, suppressed the tumor-promoting response, and improved the efficacy of anti-PD1 therapy [119,143]. Blocking the CSF-1R/CSF-1 signaling pathway by anti-CSF-1R effectively reduces the frequency and function of MDSCs in murine tumors in vivo. Importantly, CTLA-4 blockade monotherapy upregulated the expression of CSF-1R in tumor-infiltrating MDSCs and inhibited T cell proliferation, whereas anti-CSF-1R and CTLA-4 blockade combined treatment induced antitumor T-cell responses and tumor regression in multiple tumor models [144].

#### 3.2.4. Immunotherapy Combined with an Induction of Differentiation of MDSCs

Due to the high infiltration of MDSCs in solid tumors, such as breast cancer, anti-angiogenic therapies treated with anti-VEGFR2 antibodies are largely ineffective. ATRA increased the efficacy of anti-VEGFR2 antibodies alone by reversing the accumulation of MDSCs, reducing hypoxia, and secreting high levels of vessel-destabilizing S100A8 [145]. ATRA treatment in vitro reduces the immunosuppressive function of MDSCs on lymphocytes. Regarding possible mechanisms, ATRA reduces the expression of immunosuppressive genes in MDSCs, such as IDO, IL-10, NOX1, PD-L1, and TGFβ. In clinical studies, the addition of ATRA significantly reduced the level of circulating MDSCs compared with ipilimumab treatment alone in advanced-stage melanoma patients [146].

In summary, ICB monotherapy is limited and will be invalidated by the immunosuppressive effect mediated by MDSCs. Dual or triple therapy targeting MDSCs and ICB may become the focus of future cancer treatment research. Several clinical trials of the combined treatment are listed in Table 1.

### 3.3. Other Therapy Strategies

**Figure 5:**
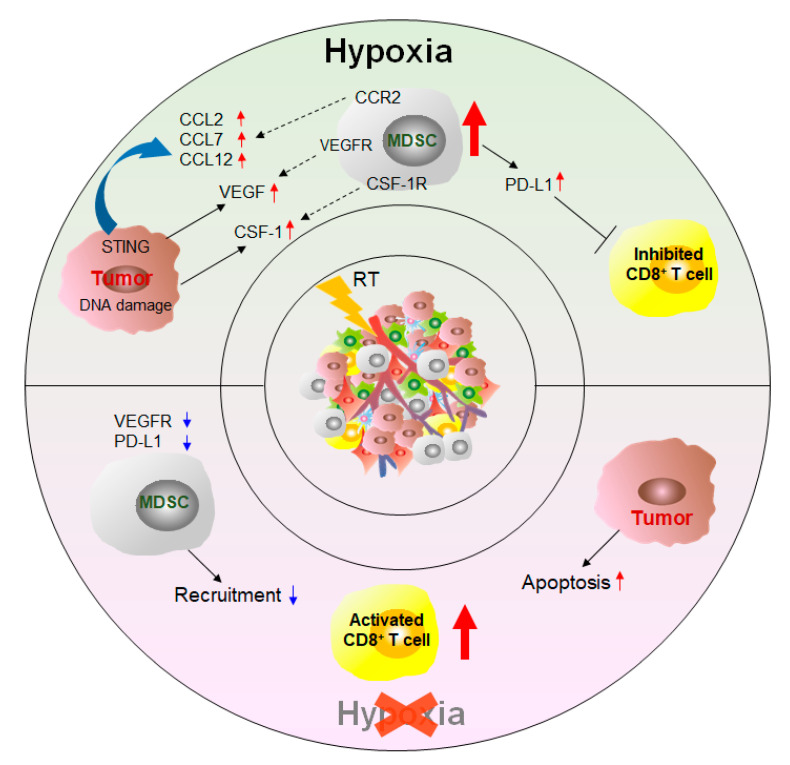
Effect of radiation therapy (RT) on MDSCs. Treatment with conventional fractionated radiotherapy (CFRT) promotes the secretion of tumor cytokines and chemokines in response to activation of the STING signaling pathway and DNA damage. These secreted factors bind to the receptors on the membrane of MDSCs, which increases the number of MDSCs migrating to the TME, upregulates the expression of PD-L1 on MDSCs, and strengthens the ability to suppress T cells. Ablative hypofractionated radiotherapy (ABHRT) reduces the recruitment of MDSCs by destroying the hypoxic environment in the TME and induces tumor apoptosis, leading to reactivation of the antitumor response. CCL, CC chemokine ligand; STING, stimulator of interferon genes; VEGF, vascular endothelial growth factor.

In addition to the targeted MDSC approaches, other treatments can affect the number and function of MDSCs and thus achieve the purpose of inhibiting tumor growth and improving survival (Figure 5).

#### 3.3.1. Radioactive Therapy

Radiotherapy is a traditional and effective method of treating cancer. Exposure of the tumor site to different doses of radiation promotes tumor cell necrosis [69]. In a mouse model of prostate cancer, exposure to conventional fractional radiotherapy (CFRT) increases the number of MDSCs in the spleen, lymph nodes, and peripheral blood as well as the level of CSF-1 by two-fold in tumors [147] (Figure 5). Further investigation has demonstrated that this effect is attributed the DNA damage induced by radiotherapy, which induces the transfer of the kinase ABL1 to the nucleus. ABL1 combines with the promoter of the CSF-1 gene to enhance the transcription of the CSF-1 gene. The increase in circulating CSF-1 promotes the infiltration of MDSCs to tumor sites through the CSF-1/CSF-1R signaling pathway. Compared to radiotherapy alone, selective inhibition of CSF-1R is more effective in assisting radiotherapy to inhibit tumor development. Radiotherapy-mediated activation of the stimulator of interferon genes (STING)/IFNγ pathway also contributes to the recruitment of MDSCs. After local ablative radiation, the STING/IFNγ pathway enhances tumor radioresistance by inducing the expression of CCL2, CCL7, and CCL12, which attract CCR2^+^ MDSCs into the TME [148]. These effects are abrogated by combination treatment with anti-CCR2 antibodies and radiotherapy [148].

Compared to CFRT, ablative hypofractionated radiotherapy (AHFRT) significantly reduces the level of MDSCs in the TME and reduces their PD-L1 expression while reducing the level of VEGF in the TME, inhibiting the VEGF/VEGFR pathway to impede the migration of MDSCs [149]. IDO1 plays an important role in the immunosuppression mediated by MDSCs. An interesting study demonstrated that IDO1 inhibition overcomes immune suppression and makes tumors sensitive to AHFRT by reducing the number of IDO1-expressing MDSCs [150]. The strategy of radiotherapy combined with anti-PD-L1 can effectively reduce the accumulation of MDSCs and remove the antitumor limitation of T cells in tumor-bearing mice and patients with nonsmall cell lung cancer [151,152]. Stereotactic body radiotherapy (SBRT) is an emerging treatment that directly and safely induces tumor cell death by directly irradiating the tumor site. Three days after SBRT, the number of MDSCs in the peripheral blood of cancer patients will be significantly reduced [153]. Sunitinib treatment promotes the therapeutic effect of SBRT, abolishes the immunosuppression of MDSCs and T_regs_, and strengthens antitumor immunity [154]. Several clinical trials of radiotherapy are listed in Table 2.

#### 3.3.2. Epigenetic Therapy

Epigenetic therapy is an emerging method of targeting MDSCs to treat cancer. Current epigenetic therapeutic approaches mainly include treatment with histone methyltransferase inhibitors (HMTis), histone deacetylase inhibitors (HDACis), and DNA methyltransferase inhibitors (DNMTis) [155]. Enhancer of zeste homolog 2 (EZH2), a gene encoding histone methyltransferase, is often overexpressed in multiple cancer types [156]. After treatment with the EZH2 inhibitor GSK343, the number of functional MDSCs increased significantly in colorectal cancer mouse models or in vitro [156]. Similarly, the use of another inhibitor, GSK126, also promoted the proliferation of MDSCs. Anti-Gr1 antibody or gemcitabine/5-FU combined with GSK126 can relieve the immunosuppression of MDSCs and increase the number of tumor-infiltrating T cells [157]. HDAC2 silences the transcription of the retinoblastoma (Rb) gene through epigenetic modification; thus, M-MDSCs acquire partial phenotypes and functions of PMN-MDSCs in tumor-bearing mice [158]. DNMTi 5-azacytidine (AZA) increases the proportion of CD8^+^ T cells and NK cells in the TME through a type I IFN immune response, reduces the accumulation of MDSCs, and promotes antitumor effects [159]. The addition of an HDACi entinostat (ENT) to AZA further enhances the regulation of the immune microenvironment. Triple or quadruple treatment of AZA and ENT plus immunotherapy anti-PD-1 and anti-CTLA-4 exhibited highly effective tumor elimination [8,159,160,161,162]. Adjuvant epigenetic therapy with AZA and ENT blocks the migration of MDSCs by downregulating CCR2 and CXCR2, which leads to the differentiation of MDSCs into macrophages and disturbance of pMN [161,163,164]. Several clinical trials of the combination of epigenetic therapy with immunotherapy are listed in Table 3.

## 4. Conclusions

Overall, MDSCs are one of the main promoters of cancer. MDSCs abolish the antitumor response by exerting immunosuppressive functions, promote the formation of the tumor microenvironment, and provide comfortable conditions for tumor growth. At present, research on MDSCs remains insufficient, and how to distinguish MDSCs from other myeloid cells remains controversial. Emerging high-throughput technologies may help to better identify the phenotype of MDSCs. Therapeutic methods targeting MDSCs have been shown to effectively limit the accumulation of MDSCs in tumor tissue and peripheral organs. In the future, the combination of targeted MDSCs and immunotherapy may become the main cancer treatment strategy.

## Figures and Tables

**Table 1:** Clinical trials of the regulation of MDSCs.

Strategy	Target	Intervention	Drug	Cancer Types	Clinical Trial	Status
Inhibition of suppressive functions	COX-2	Celecoxib	Nivolumab Ipilimumab	Colon carcinoma	NCT03026140	Recruiting
	PDE5	Tadalafil Placebo	N/A	Head and neck squamous cell carcinoma	NCT01697800	Completed
	STAT3	BBI608	Nivolumab	Colorectal cancer	NCT03647839	Recruiting
	ARG1	ARG1 peptide	PD-L1 peptide	Myeloproliferative neoplasms	NCT04051307	Recruiting
	VEGF	Bevacizumab	Capecitabine	Breast cancer	NCT00109239	Completed
	IDO	Indoximod	Gemcitabine	Pancreatic cancer	NCT02077881	Completed
	BTK	Lbrutinib	Nivolumab	Renal cell	NCT02899078	Recruiting
	Androgen	Androgen-ablation therapy	Cabozantinib	Prostate cancer	NCT01630590	Active
	Estrogen	Anti-estrogen therapy	Afinitor	Breast cancer	NCT02291913	Completed
Depletion	N/A	5-FU	Gemcitabine Cisplatin	Pancreatic cancer Biliary cancer	NCT01661114	Completed
	N/A	Docetaxel	Bevacizumab	Breast cancer	NCT00217672	Completed
	LXR	RGX-104	Nivolumab Ipilimumab	Malignant neoplasms	NCT02922764	Recruiting
	BET bromodomain	GSK525762	Placebo Fulvestrant	Neoplasms	NCT02964507	Active
Blockade of migration	CXCR2	SX-682	Pembrolizumab	Melanoma	NCT03161431	Recruiting
	CXCR4	BL-8040	Pembrolizumab	Pancreatic adenocarcinoma	NCT02907099	Active
	CCR5	Maraviroc	Pembrolizumab	Colorectal Cancer	NCT03274804	Completed
	CSF-1R	Pexidartinib	Durvalumab	Colorectal cancer Pancreatic cancer Metastatic cancer	NCT02777710	Completed
Induction of differentiation	RAR/RXR	ATRA	Pembrolizumab	Melanoma	NCT03200847	Recruiting
		Vitamin D3	Bevacizumab chemotherapy	Colorectal adenocarcinoma	NCT04094688	Recruiting

**Table 2:** Clinical trials of radiation therapy.

Study Title	Radiotherapy	Drug	Cancer Types	Clinical Trial	Status
Trial of SBRT with concurrent Ipilimumab in metastatic melanoma	SBRT 24 Gy in 8 fractions 30 Gy in 10 fractions 36 Gy in 12 fractions	Lpilimumab	Melanoma	NCT02406183	Completed
Atezolizumab with stereotactic ablative radiotherapy in patients with metastatic tumors	SBRT 45 Gy in 3 fractions	Atezolizumab	Colorectal cancer Non-small lung cancer Renal cell carcinoma Sarcoma	NCT02992912	Recruiting
Anti-PD-1 ± RT for MSI-H solid tumors	Regimen not stated	Anti-PD-1	Colorectal cancer	NCT04001101	Recruiting
Preoperative Radiotherapy and Chemotherapy in patients with locally advanced rectal cancer (PROArCT)	25.2 Gy in 14 fractions	Oxaliplatin Fluorouracil Leucovorin	Rectal cancer	NCT01013805	Completed
Pembrolizumab in muscle invasive/metastatic bladder cancer (PLUMMB)	24 Gy in 6 fractions		Bladder cancer	NCT02560636	Active

**Table 3:** Clinical trials of epigenetic therapy combined with immunotherapy.

Target	Intervention	Drug	Cancer Types	Clinical Trial	Status
EZH2	Tazemetostat	Pembrolizumab	Urothelial carcinoma	NCT03854474	Recruiting
EZH2	CPI-1205	Ipilimumab	Advanced solid tumors	NCT03525795	Active
HDAC	Panobinostat	Ipilimumab	Melanoma Skin cancer	NCT02032810	Active
HDAC6	ACY-241	Nivolumab	Non-small cell lung cancer	NCT02635061	Active
HDAC	Panobinostat	Ipilimumab	Melanoma Skin cancer	NCT02032810	Active
HDAC	Mocetinostat	Durvalumab	Advanced cancer	NCT02805660	Completed
HDAC	Mocetinostat	Pembrolizumab Guadecitabine	Lung cancer	NCT03220477	Recruiting
DNMT	Decitabine	Nivolumab Tetrahydrouridine	Lung cancer non-small cell lung cancer	NCT02664181	Completed
DNMT	5-Azacitidine	Entinostat Nivolumab	Non-small lung cancer	NCT01928576	Recruiting
	Azacitidine	Oxaliplatin Epirubicin Capecitabine	Esophageal cancer	NCT01386346	Completed
DNMT	Temozolomide	Nivolumab	Brain cancer	NCT02617589	Active
DNMT	Guadecitabine	Nivolumab	Colorectal carcinoma	NCT03576963	Recruiting
DNMT	Entinostat	Pembrolizumab	Myelodysplastic Syndrome	NCT02936752	Recruiting
